# Computational Methods for Single-cell Multi-omics Integration and Alignment

**DOI:** 10.1016/j.gpb.2022.11.013

**Published:** 2022-12-26

**Authors:** Stefan Stanojevic, Yijun Li, Aleksandar Ristivojevic, Lana X. Garmire

**Affiliations:** 1 Department of Computational Medicine and Bioinformatics, University of Michigan, Ann Arbor, MI 48109, USA; 2 Department of Biostatistics, University of Michigan, Ann Arbor, MI 48109, USA; 3 Amherst College, Amherst, MA 01002, USA

**Keywords:** Single-cell, Multi-omics, Machine learning, Unsupervised learning, Integration

## Abstract

Recently developed technologies to generate **single-cell** genomic data have made a revolutionary impact in the field of biology. **Multi-omics** assays offer even greater opportunities to understand cellular states and biological processes. The problem of integrating different omics data with very different dimensionality and statistical properties remains, however, quite challenging. A growing body of computational tools is being developed for this task, leveraging ideas ranging from machine translation to the theory of networks, and represents another frontier on the interface of biology and data science. Our goal in this review is to provide a comprehensive, up-to-date survey of computational techniques for the **integration** of single-cell multi-omics data, while making the concepts behind each algorithm approachable to a non-expert audience.

## Introduction

Single-cell sequencing technologies have opened the door to investigating biological processes at an unprecedentedly high resolution. Techniques such as Drop-seq [1], InDrops [2], and 10x Genomics assays [3] are capable of measuring single-cell gene expression [single-cell RNA sequencing (scRNA-seq)] in tens of thousands of single cells simultaneously. Measurements of other data modalities are also increasingly available. For example, single-cell assay for transposase-accessible chromatin with sequencing (scATAC-seq) assesses chromatin accessibility, and single-cell bisulfite sequencing captures DNA methylation, all from single cells. However, many of such techniques are designed to measure a single modality and do not lend themselves to multi-omics measurements. The way to combine information from such measurements is then to assay different omics from different subsets of the same samples. By assuming that cells assayed by different techniques share similar properties, one can then use alignment methods to computationally aggregate similar cells across different omics assays and draw consensus biological inferences.

**Figure 1:**
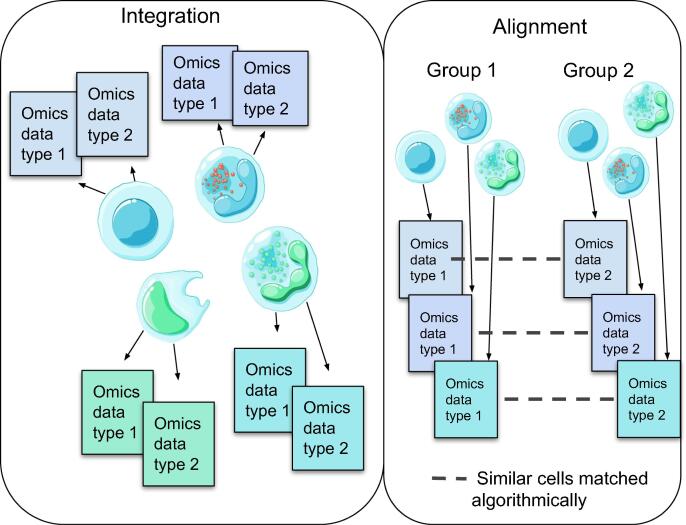
**Integration and alignment of multi-omics data** Multi-omics data can sometimes be sequenced from the same set of single cells (left); at other times, only the data sequenced from the same/similar sample, but different single cells are available (right). In the former case, we have the task of integrating the different data modalities (left); in the latter case, we need to first identify similar cells across the samples (right). This is the computational task of alignment.

Recently, however, a number of experimental techniques capable of assaying multiple modalities simultaneously from the same set of single cells have been developed. Cellular indexing of transcriptomes and epitopes by sequencing (CITE-seq) [4] and RNA expression and protein sequencing (REAP-seq) [5] measure protein and gene expression. Single-nucleus chromatin accessibility and mRNA expression sequencing (SNARE-seq) [5], [6], simultaneous high-throughput ATAC and RNA expression with sequencing (SHARE-seq) [7], and single-cell combinatorial profiling of chromatin accessibility and mRNA (sci-CAR) [8] measure gene expression and chromatin accessibility, while single-cell sequencing of gene expression and methylation (scGEM) [9] measures gene expression and DNA methylation and genome and transcriptome sequencing (G&T-seq) [10] assays genome and transcriptome. For triple-omics data generation, single-cell nucleosome, methylation, and transcription sequencing (scNMT) [11] measures gene expression, chromatin accessibility, and DNA methylation, while single-cell triple-omics sequencing (scTrio-seq) [9], [12] captures single nucleotide polymorphisms (SNPs), gene expression, and DNA methylation simultaneously. The multiome platform by 10x Genomics is capable of measuring gene expression and chromatin accessibility at the same time. Integrative analysis of such data obtained from the same cells remains a challenging computational task due to a combination of reasons, such as the noise and sparsity in the assays, as well as different statistical distributions for different modalities. For clarity, we distinguish between integration methods that combine multi-omics data from the set of the same single cells, from alignment methods designed to work with multi-modal data coming from the same tissue but different cells. The difference in their approaches is shown in Figure 1.

The application of data fusion algorithms for multi-omics sequencing data predates single-cell technologies; bulk-level data have been integrated using a variety of computational tools as reviewed previously [13]. In this review, we aim to give a comprehensive, up-to-date summary of existing computational tools of multi-omics data integration and alignment in the single-cell field, for researchers in the field of computational biology. For more general surveys, the readers are encouraged to check other single-cell multi-omics reviews [14], [15], [16], [17], [18], [19], [20], [21]. Distinctively, the targeted readers of our review are computational biologists trying to understand computational tools at a detailed technical level. Therefore, the work here covers the fundamental principles of underlying algorithms in-depth and elaborates on the strength and weaknesses of these approaches whenever applicable.

## Integration methods handling multi-omics data generated from the same single cells

**Figure 2:**
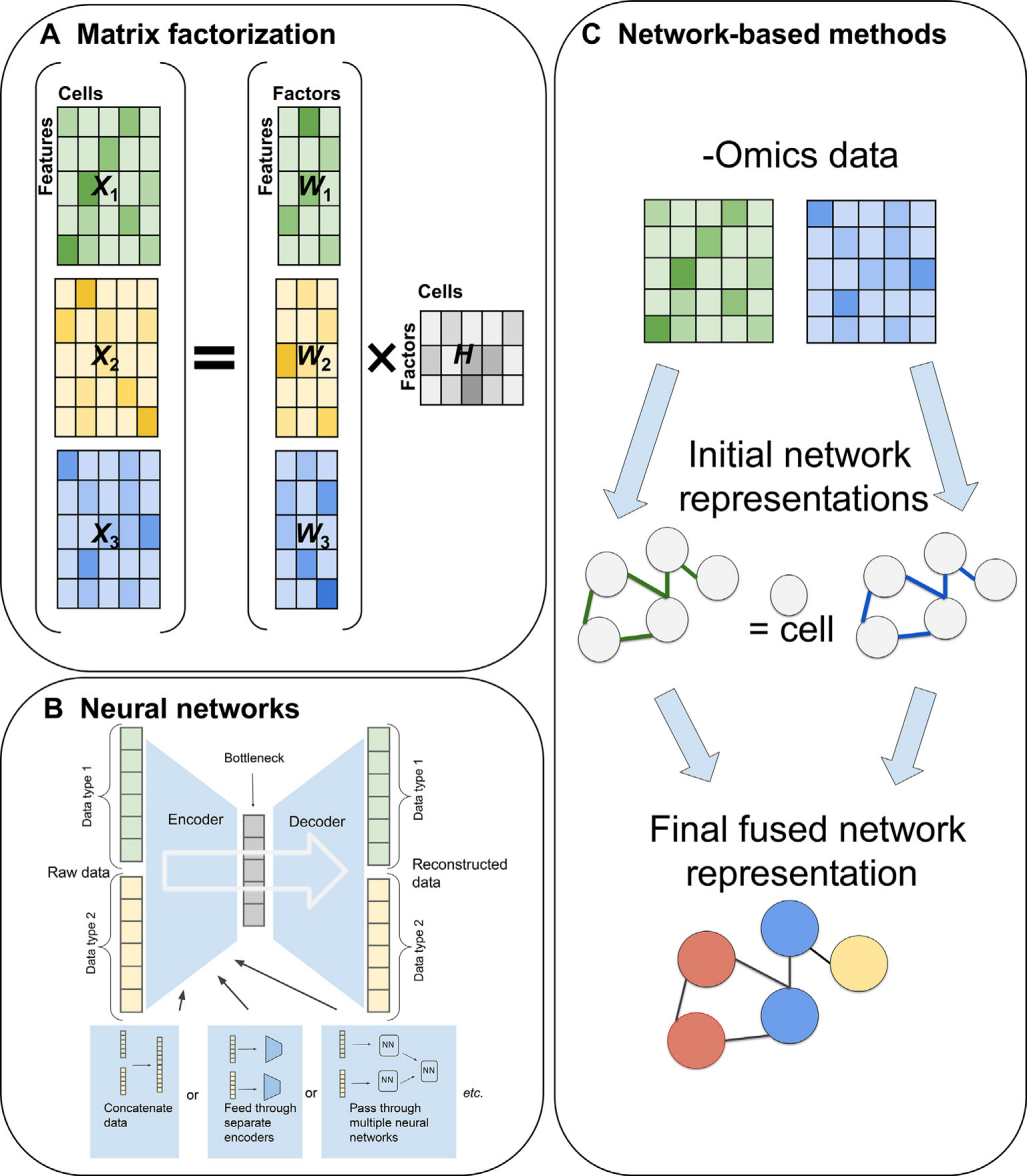
**Single-cell multi-omics integration methods** Illustration of some common integration approaches for single-cell multi-omics data: matrix factorization uncovering a representation of both cells and omics via factors (**A**), neural networks which combine different -omics into a single cell representation (**B**), and network-based approaches, which represent cells as nodes on the graphs connected to nearby cells (**C**).

The integration methods for multi-modal data assayed from the same set of single cells can be conceptualized as “vertical integration”, which was mentioned in an earlier review [17], can be broadly categorized into at least three main types by methodology: mathematical matrix factorization-based methods, artificial intelligence (AI; *e.g.*, neural network)-based methods, and network-based methods. The scheme of these methods is illustrated in Figure 2. Additional less diversified approaches include a Bayesian statistical method and a metric learning method. The list of the currently implemented methods is summarized in Table 1 and Table 2.

**Table 1:** **Summary of the methods for integrating multi-omics data from the same cells**

**Methodology category**	**Method**	**Algorithm**	**Data**	**Advantageand disadvantage**	**Ref.**
Matrix factorization	MOFA+	Matrix factorization with automatic relevance determination	Transcriptomic, epigenetic	• GPU enables scalability to millions of cells • MOFA+ can only capture moderate non-linear relationships	[7]
	scAI	Pseudotime reconstruction and manifold alignment	Transcriptomic, epigenetic	• Sensitive enough to capture cell states when only one mode of data is distinct across cell states • scAI’s missing value strategy cannot impute missing values or distinguish between methylated and missing states for DNA methylation data	[10]
Neural network	scMVAE	Variational autoencoder	Transcriptomic, epigenetic	• The scMVAE framework is flexible to encompass diverse joint-learning strategy • No guiding principles are provided with respect to how to pick a specific learning strategy for the specific dataset	[27]
	DCCA	Variational autoencoder	Transcriptomic, epigenetic	• Able to generate biologically meaningful missing omics data based on the learned latent representation of another omics data • Performance is not robust against high levels of noise	[30]
	totalVI	Variational autoencoder	Transcriptomic, proteomic	• Computationally scalable and flexible	[12]
	LIBRA	Split-brain autoencoder	Transcriptomic, proteomic, epigenetic	• Computationally scalable • Depending on the dataset, extensive fine-tuning may be required to achieve optimal performance • Does not explicitly deal with missing data	[32]
	BABEL	Autoencoder translating between modalities	Transcriptomic, proteomic, epigenetic	• BABEL’s autoencoder model follows an efficient interoperable design, resulting in efficient cross-modality prediction • BABEL’s performance is limited by the amount of mutual information shared between the input data modalities	[34]
	DeepMAPS	Graph neural network	Transcriptomic, epigenetic, proteomic	• Learns interpretable cell type-specific biological networks based on data modality • Computational cost does not scale efficiently to super-larger datasets • Reproducibility could be dependent on the specific GPU model	[35]
Network-based	citeFUSE	Similarity network fusion	Transcriptomic, proteomic	• Enables doublet detection • Computationally scalable • Performance could be dependent on the structure of the input modality graphs	[36]
	Joint diffusion	Joint manifold learning through Integrated diffusion	Transcriptomic, epigenetic	• Enables simultaneous denoising of input datasets • Has not been tested on enough real datasets, therefore the robustness of its performance remains to be seen	[39]
	Seurat v4	Weighted averaging of nearest neighbor graphs	Transcriptomic, proteomic	• The modality weights learned by WNN are interpretable as the representation of technical quality and importance of modality measurement • Requires dimension reduction, which is not compatible with categorical or binary input	[41]
Others	BREM-SC	Bayesian mixture model	Transcriptomic, proteomic	• Enables quantification of clustering uncertainty • Explicitly addresses the between-modality correlation • The MCMC algorithm can be computationally expensive	[42]
	SCHEMA	Metric learning	Transcriptomic, epigenetic	• Computationally efficient • Performance and interpretability may be affected by the choice of primary modality	[43]

*Note*: MOFA, multi-omics factor analysis; scAI, single-cell aggregation and inference; scMVAE, single-cell multimodal variational autoencoder; DCCA, deep cross-omics cycle attention.

**Table 2:** **Extended summary of the methods for integrating multi-omics data from the same cells**

**Method**	**Programming language**	**Link**	**GEO accessionof datasets tested on**
MOFA+	Python, R	GitHub	GSE87038, GSE97179, GSE121708
scAI	R, MATLAB	GitHub (R), GitHub (MATLAB)	GSM3271044, GSM3271045, GSM3271040, GSM3271041, GSE74535
ScMVAE	Python	GitHub	GSE126074
DCCA	Python	GitHub	GSE126074, GSE140203, GSE109262
totalVI	Python	Package webpage	GSE150599, PBMC5k, PBMC10k, MALT
LIBRA	Python, R	GitHub	GSE126074, GSE128639, GSE130399, GSE140203, SE194122, GSE109262, PBMC10k (ATAC-seq), PBMC10k (scRNA-seq), Human Bone Marrow
BABEL	Python	GitHub	GSE160148, PBMC10k, GSE166797, GSE126074, GSE140203
DeepMAPS	Python	GitHub	GSE84133, GSE128639, GSE148665, GSE100866, GSE121708, PBMC10k, GSE150599, PBMC3k, PBMC3k_filtered, PBMC10k_filtered, fresh_embryonic_E18_mouse_brain, human_brain, PBMC10k_multiome, SRP136421, PBMC_or_lung_tumor_leukocytes, human_lymph_node
citeFUSE	R	Bioconductor	GSE126310
Joint diffusion			
Seurat v4	R	Package webpage	GSE164378, GSE100866, GSE128639, GSE156473, PBMC10k_multiome, GSE140203, PBMC_citeseq, PBMC_scrnaseq
BREM-SC	R	GitHub	GSE148665, PBMC10k
SCHEMA	Python	GitHub	Slide-Seq, GSE117089, ENCFF336WPU, T cell motif data, GSE107451, mouse gastrulation dataset, GSE95753

### Matrix factorization-based methods

Matrix factorization-based methods aim to describe each cell as the product between a vector that describes each omics element (genes, epigenetic loci, and proteins) and a vector of reduced and common features (factors) capturing its basic properties (Figure 2A). Mathematically, if we represent each omics as matrix *X*_*i* (*i*=1,2__,__..__.__)_ then matrix factorization decomposes it as the product of a shared matrix *H* across all omics data types, and omics-specific matrix *W*_*i* (*i*=1,2__,__..__.__)_, together with random noise *ε*_*i* (*i*=1,2,…)_ as:

(1) $$X_{1}=W_{1}\;H+\varepsilon_{1\;},\;X_{2}=W_{2}\;H+\varepsilon_{2}\;,\;\cdots \;,\;\;X_{i}=W_{i}\;H+\varepsilon_{i}$$

Such methods are simple and easily interpretable since the cell and omics factors can be associated with omics features, but may lack the ability to capture nonlinear effects. We describe the variations in this type of method below. MOFA+ [22] is a sequel to the multi-omics factor analysis (MOFA) [22], [23]. Both studies perform factor analysis, equipped with sparsity-inducing Bayesian elements including automatic relevance determination [24]. MOFA+ integrates data over both views (corresponding to different modalities) and groups (corresponding to different experimental conditions). The model scales easily to large datasets. MOFA+ was applied to integrate gene expression, chromatin accessibility, and DNA methylation data assayed using scNMT from mouse embryos, as well as to integrate several datasets over different experimental conditions rather than different omics. After performing factor analysis on the mouse dataset, the most relevant factors are related to biological processes shaping embryo development. MOFA+ provides an elegant and successful general framework for integration, which could potentially be superseded in specific cases by more specialized models designed for integrating specific omics layers.

Single-cell aggregation and inference (scAI) [25] features a twist on matrix factorization and is designed specifically for the integration of epigenetic (chromatin accessibility and DNA methylation) and transcriptomic data. It addresses the sparsity of epigenetic data by aggregating (averaging) such data between similar cells. This requires a notion of cell–cell similarity, which is learned as a part of the model, rather than being postulated prior to the integration. Their model solves the following optimization problem:

(2) $${\min_{{}_{{}_{W_{1},\;W_{2},\;H,\;Z}}}}_{\;}\alpha ||X_{1}-W_{1}H|{|}_{F}^{2}\;+\;||X_{2}\left(Z\cdot R\right)\;-\;W_{2}H|{|}_{F}^{2}+\;\lambda ||Z\;\;-\;H^{T}H|{|}_{F}^{2}\;+\;\gamma \underset{j}{\sum }\left|\right|H_{.j\;}|{|}_{1}^{2}$$

Here *X*_1_ represents the transcriptomic data, *X*_2_ represent the epigenomic data, *H* is the common (cell-specific) factor matrix, *W*_1_ and *W*_2_ are the assay-specific factor matrices, *Z* is the cell–cell similarity matrix, and entries of *R* are Bernoulli-distributed random variables, and hyperparameters α, λ, and γ determine the relative importance of different terms. The twist on the usual matrix factorization is made by factoring aggregated epigenetic data *X*_2_ (*Z*・*R*), rather than directly factoring the epigenetic data *X*_2_. After the learning is complete, the matrix of cell factors is used to cluster the cells and the importance of genes and epigenetic marks is ranked using the magnitude of the values in loading matrices. In order to jointly visualize different factors, scAI implements a novel VscAI algorithm utilizing Sammon mappings [26]. The relationships between epigenetics and gene expression can be explored using correlation analysis and nonnegative least square regression. The model was tested on simulations using MOSim [27], and several real-world datasets, and performed better than the earlier MOFA version, in terms of identifying natural clusters and condensing epigenetic data into meaningful factors.

### Neural network-based methods

Although neural networks are generally well-suited for supervised tasks, a class of neural networks called autoencoders is commonly used for unsupervised learning, such as the multi-omics integration problem in single cells. Deep autoencoders perform nonlinear dimensionality reduction by squeezing the input through a lower-dimensional hidden layer (bottleneck) and attempting to reconstruct the original input as the output of the neural network (Figure 2B). They consist of two parts: the encoder network performing the dimensionality reduction and the decoder network reconstructing based on the dimensionally reduced data. In principle, autoencoders generalize the principal component analysis by allowing for nonlinear transformations. Many variations of autoencoder models exist, and among them, variational autoencoders have proven useful for analyzing single-cell data. Rather than directly encoding the data in a dimensionally reduced (latent) space, variational autoencoders sample from a probability distribution (usually Gaussian) in the latent space, and use the encoder network to produce the parameters of this distribution. As such, they combine deep learning and Bayesian inference to produce generative models, which not only dimensionally reduce the original data but also produce realistic synthetic data points. Below we review the methods using certain variations of the autoencoder architecture to integrate single-cell multi-omics data.

Single-cell multimodal variational autoencoder (scMVAE) [28] was designed to integrate transcriptomic and chromatin accessibility data, using a version of a variational autoencoder. The key question in multi-omics integration is how to encode the multi-omics data into a single latent space representation. In the case of scMVAE, a combination of 3 different methods was used for this task, including a neural network acting on the concatenated input data, neural networks encoding transcriptomic and chromatin accessibility data separately prior to merging, and a product of experts technique for combining different representations [29]. At the same time, cell-specific scales used to normalize expression across cells are learned (called library factors). The input data are reconstructed by processing the latent representations via decoder neural networks, which calculate the probabilities of gene dropouts and predict the expression of measured genes modeled as a negative binomial distribution.

This model incorporates the task of constructing shared representations of the multi-modal data with clustering. Namely, one of the latent variables is constructed to correspond to the cluster identifier. Furthermore, the model incorporates tools to deal with tasks such as data imputation and can be used for studying the association between epigenetics and gene expression. scMVAE was applied to integrate two real datasets assaying mRNA and chromatin accessibility using SNARE-seq method, as well as simulated data generated by Splatter [30]. It takes into account the known relationships between appropriately located transcription factors and gene expression and uses them to test the imputed (denoised) data. According to the authors, scMVAE performed better than MOFA in terms of clustering and enhancing the consistency between different -omics layers on several real and simulated datasets.

Deep cross-omics cycle attention (DCCA) model is another method in this category for joint analysis of single-cell multi-omics data [31]. It uses variational autoencoders to integrate multi-omics data and builds on the scMVAE algorithm described above. However, DCCA diverges from scMVAE in one important aspect. DCCA uses separate but coupled autoencoders to dimensionally reduce different omics layers, while scMVAE constructs a shared dimensionally reduced representation of transcriptomic and epigenetic data. This strategy is inspired by the theory of machine translation, notably the so-called attention transfer; in this case, the teacher network working with the scRNA-seq data guides the learning of the student network working with scATAC-seq data. Their model compares favorably to scAI and MOFA+ on metrics such as clustering accuracy, denoising quality, and consistency between different omics.

totalVI [32] combines Bayesian inference and a neural network to create a generative model for data integration. It was created to handle gene expression and protein data. Joint latent space representations are learned via an encoder network and used to reconstruct the original data while accounting for the difference between the original data modalities. The model generates latent representations capturing both omics, and at the same time models experimental conditions through an additional set of latent variables. The gene expression data are sampled from a negative binomial distribution, and the parameters are obtained as outputs of a decoder neural network. The protein data are sampled from a mixture model with two negative binomial distributions simulating the experimental background and the actual signal, respectively. The model was applied to two datasets containing transcriptomic and proteomic measurements and generated shared representations of cells with interpretable components.

LIBRA [33] uses an autoencoder-like neural network to translate between different omics. Motivated by split-brain autoencoder [34] and machine translation approach, the model consists of two separate neural networks. The first network takes as input elements of the first dataset and aims to reconstruct a corresponding element of the second dataset. The second network performs an inverse task. Taken together, the bottlenecks of two networks aim to convert the two datasets into the same latent space. This method is quite general and can be applied to various pairs of omics data. It produced clusters of similar quality compared to Seurat v4.

BABEL [35] also uses autoencoder-like neural networks to translate between gene expression (modeled by negative binomial distribution) and binarized chromatin accessibility data. There are two encoder and two decoder neural networks, each encoder/decoder handles one data type of gene expression or chromatin accessibility. As a result, four combinations between encoders and decoders are formed, and the loss function is optimized to minimize reconstruction error for four combinations of encoders and decoders. In this approach, the two encoders are prone to produce similar representations, as the encoded gene accessibility is decoded as chromatin accessibility and *vice versa*.

BABEL provides a promising generic framework for multi-omics inference at a single-cell level from single-omics data, by using the model that was previously trained on multi-omics data sequenced from the same single cells. The modular nature of BABEL provides additional flexibility, as the model can be extended to work with additional modalities when the corresponding data becomes available. Despite the potential for generalization, one should be cautioned that if the training is conducted on cell types that are very different, the transfer learning using BABEL is not very successful.

DeepMAPS [36] integrates different data modalities by a graph transformer neural network architecture for interpretable representation learning. The data are represented using a heterogenous graph in which some of the nodes represent cells and others represent genes. An autoencoder-like graph neural network architecture is used for representation learning, with an attention mechanism. The attention mechanism learns the weights by the contribution of the neighbors to the node of interest. This not only achieves better performance, but also enhances the interpretability to identify genes most relevant to cell state differences. DeepMAPS method learns relevant gene-gene interaction networks and cell–cell similarities, which can be used for downstream steps such as clustering to infer novel cell types. It compared favorably on clustering, compared to state-of-the-art techniques such as MOFA+ and totalVI.

### Network-based methods

Network-based methods represent the relationships between different cells using a weighted graph, where cells serve as nodes (Figure 2C). Integration is then accomplished by manipulating such graph representation. This approach emphasizes the neighborhood structure and sometimes pools the information between neighbors, leading to additional robustness against the noise. Below are the currently available methods.

citeFUSE [37] integrates transcriptomic and proteomic CITE-seq data using network fusion of similarity graphs corresponding to different modalities. This idea traces back to computer science work [38] on fusing multi-view networks through cross-diffusion, and to the follow-up SNF method [39] that was used to integrate bulk-level multi-omics data. The algorithm adjusts the graph connectivities by a process of diffusion, which allows for the distance information to be aggregated between neighbors. Namely, the algorithm consists of two iterative steps: separate diffusion on different -omics layers and fusion across the omics layers. It results in a fused consensus matrix of distances between cells, borrowing information from multiple omics. citeFUSE used spectral clustering to identify cell types and showed an improvement over single-modality-based clusters. Additional benefits of the method include inference of ligand-receptor interactions and a novel tool for doublet detection.

Joint diffusion [40] constructs graph representations of different -omics and then performs a joint diffusion process on the two graphs in order to denoise and integrate the data. This approach builds upon MAGIC [41], a method for denoising scRNA-seq data, and generalizes it to multi-modal data. Diffusion can be conceptualized as a random walk process. In the graph diffusion algorithm, random walking on the graph can help discover the intrinsic structure of the data hidden behind the noise. In joint diffusion, random walks are performed while allowing for transitions from one graph to another. A key idea in this work is to quantify the amount of noise in different datasets, through a spectral entropy of the corresponding graphs, and adjust the time one spends on different graphs in accordance with their relative levels of noise. In this way, the transcriptomic and epigenetic data will not be weighted equally, as the transcriptomic data are generally of better quality. This method excels at denoising and visualizations and was shown to present an improved clustering performance compared to single-modality clustering and the one based on a more naive alternating diffusion process.

Seurat v4 [42] aims to represent the data as a weighted nearest neighbor (WNN) graph in which cells that are similar according to the consensus of both modalities are connected. In the process of constructing a WNN graph, a set of cell-specific weights dictating the relative importance of different omics data is learned. Such weights often carry important biological meanings. Specifically, Seurat v4 pipeline has the following steps: first, data corresponding to different omics are dimensionally reduced using principal component analysis (PCA) to the same number of dimensions. Then, k-nearest neighbor (kNN) graphs corresponding to different omics are constructed. In a kNN graph, each datapoint (a node of this graph) is connected to *k* nearest neighboring nodes. Cell-specific coefficients determining the relative importance of different omics are then learned by considering the accuracy of inter-modality and cross-modality predictions by nearest neighbor graphs. Lastly, a linear combination of data from different omics is done, using the coefficients learned in the previous step. The nearest neighbors with respect to those linear combinations are then connected to build the WNN graph. Seurat v4 was applied to a CITE-seq-based transcriptomic and proteomic dataset, and several other datasets involving mRNA, proteins, and chromatin accessibility. The authors compared this method with MOFA+ and totalVI, using correlations (Pearson and Spearman) between the data corresponding to a cell and the average of its nearest latent space neighbors, and claimed that it performed better than MOFA+ or totalVI.

### Other models

BREM-SC [43] is a Bayesian mixture method. It integrates single-cell gene expression and protein data by modeling them as a mixture of probability distributions that share the same underlying set of parameters. The model is useful for performing joint clustering, where confidence in cluster assignments can be quantified via posterior probabilities. It performed favorably compared to single-omics clustering methods. Although the Markov Chain Monte Carlo (MCMC) procedure used to train the model can be computationally intensive, the model provides an effective way of integration by accounting for the differences between the two omics layers using probability distributions.

SCHEMA [44] is a different metric learning approach that aims to construct a notion of distances on the space of samples, taking into account different omics data. One of the omics (usually, scRNA-seq) is considered the primary base for distance, additional omics are then used to modify this distance. This is formulated as optimization of the quadratic function using quadratic programming. The scRNA-seq and scATAC-seq data can thus be integrated, yielding downstream insights into cell developmental trajectories. This method showed a better clustering performance than those based on clustering different modalities separately or integrating them using canonical correlation analysis. It is a useful method for asymmetrically integrating data modalities of different qualities, such as the case of scRNA-seq and scATAC-seq data.

## Alignment methods handling multiple genomics data generated from different single cells of the same tissue

**Figure 3:**
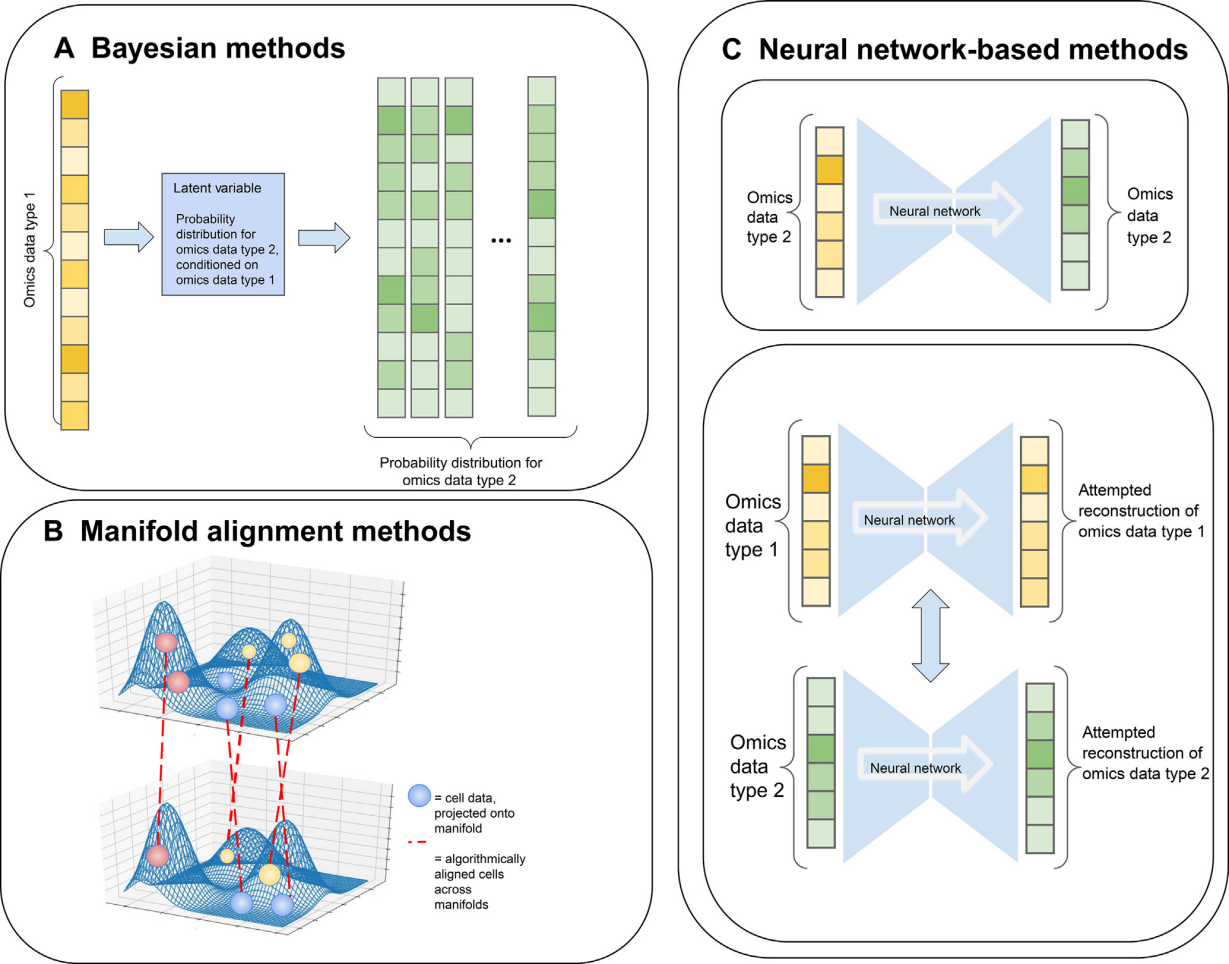
**Single-cell multi-omics alignment methods** Illustration of some common approaches for alignment of multi-omics single-cell data: Bayesian methods, modeling the probability distribution of -omics measurements using a number of latent variables and updating such distributions using Bayes’ formula (**A**), manifold alignment methods uncovering a surface in the space of omics on which the alignment can be performed (**B**), and neural network-based models, creating latent representations of different -omics data, which can be more easily aligned (**C**).

Compared to multi-omics data, it is experimentally much easier to obtain multiple modalities of data where each modality is obtained from similar but different cells of the same tissue. The task to harmonize these data is called alignment (Figure 1), and it is synonymous with diagonal integration as described in another earlier review [17]. The body of literature applying machine learning and statistical methods to this task is rich, including manifold learning, neural network-based methods, and Bayesian methods, as summarized in Table 3 and Table 4 and depicted in Figure 3. It is important to note that in multi-omics we do not know priorly the cell correspondences across omics layers, therefore besides the effort on learning representations of the cells in the multi-omics integration described in the previous section, additional attention needs to be paid to align the distributions of these representations. As a result, methods designed for integration are generally not capable of doing the alignment. Conversely, methods designed for alignment may perform sub-optimally for integration tasks.

**Table 3:** **Summary of the methods for aligning multi-omics data from the same cells**

**Methodology category**	**Method**	**Algorithm**	**Data**	**Advantageand disadvantage**	**Ref.**
Bayesian	Clonealign	Bayesian latent variable model	RNA-seq, DNA		[45]
	MUSIC	Topic models	RNA, CRISPR		[46]
Manifold alignment	MATCHER	Pseudotime reconstruction and manifold alignment	Transcriptomic, epigenetic	• Finds pseudotime trajectories while performing alignment • Assuming a non-branching pseudotime trajectory	[51]
	MMD-MA	Manifold alignment	Transcriptomic, epigenetic (DNAme)	• Robust with respect to hyperparameters • Assuming that two datasets contain the similar distribution of cells	[53]
	UNION-Com	Topological alignment	Transcriptomic, epigenetic	• Aligning without requiring identical distributions of cells	[48]
	SCOT	Gromov-Wasserstein optimal transport	Transcriptomic, epigenetic (DNAme)	• Computational efficiency, automated hyperparameter tuning	[49]
	Pamona	Partial Gromov-Wasserstein optimal transport	Transcriptomic, epigenetic	• Designed for aligning unbalanced datasets	[50]
Neural network	SCIM	Adversarial autoencoder	Transcriptomic, proteomic (CyTOF)	• Allows for complicated nonlinear mappings into latent space • Assuming that the two datasets contain the similar distribution of cells	[60]
	Multigrate	Variational autoencoder	Transcriptomic, proteomic	• Allows for mapping onto a multi-modal atlas • Assuming that the two datasets contain the similar distribution of cells	[63]
	MAGAN	Generative adversarial network	Transcriptomic, proteomic	• Implements a twist to incentivize correct mapping of cell types, not just distributions • Needs some correspondence information for best performance	[64]
Others	Seurat v3	Canonical correlation analysis and mutual nearest neighbors analysis	RNA-seq, ATAC-seq	• Popular, widely used, and comprehensive package • Useful for multi-omics alignment, but created for aligning scRNA-seq datasets	[66]
	MAESTRO	Canonical correlation analysis	RNA-seq, ATAC-seq	• Provides alignment as a part of a comprehensive analysis pipeline • Uses a generally applicable algorithm for integration, not optimized for multi-omics	[67]
	bindSC		RNA-seq, ATAC-seq	• Generates latent representations while aligning datasets	[68]
	LIGER	Matrix factorization	RNA-seq, methylation	• Computationally efficient • Useful for multi-omics alignment, but created for aligning scRNA-seq datasets	[69]

*Note*: CRISPR, clustered regularly interspaced short palindromic repeats; DNAme, DNA methylation; CyTOF, cytometry by time of flight.

**Table 4:** **Extended summary of the methods for aligning multi-omics data from the same cells**

**Method**	**Programming language**	**Link**	**Datasettested on**
Clonealign	R	GitHub	EGAD00001004552, EGAD00001004553, EGAS00001002170
MUSIC	R	GitHub	E-MTAB-5061, E-MTAB-5060 , GSE81608, GSE81433, GSE50244, GSE107585, GSE81492, GSE56743, GSE65267, GSE79443
MATCHER	Python	GitHub, documentation	E-MTAB-2600, GSE70253, GSE74535, GSE56879
MMD-MA	Python	Code and data	Data
UNION-Com	Python	GitHub	Data supplement
SCOT	Python	Package webpage	SRP077853, GSE126074
Pamona	Python	GitHub	Dataset, GSE121708, GSE126074
SCIM	Python	GitHub	SCIM dataset
Multigrate	Python	GitHub	GSE47353, GSE41080, GSE59654, GSE59743, GSE29619, GSE74817, GSE13486, GSE65391, GSE164378, GSE128639, GSE156473, GSE140203
MAGAN	Python	GitHub	GSE75478, GSE72857
Seurat v3	R	Package webpage, GitHub	GSE164378,GSE100866, GSE128639, GSE156473, GSE140203
MAESTRO	R, Python	Package website, GitHub	GSE65360, GSE74310, GSE96772, GSE123814, GSE129785
bindSC	R	GitHub	GSE201402, GSE190976
LIGER	R	GitHub	GSE92495, GSE116470, GSE126836

### Bayesian methods

Clonealign [45] integrates single-cell RNA and DNA sequencing data from heterogeneous populations by assigning cells measured by RNA-seq to clones derived from DNA-seq data. Clonealign is based on a Bayesian latent variable model, where a categorical variable is used to specify cell assignment. The model maps the copy number of a gene to its expression value by introducing a copy number dosage effect on the gene expression. The model is also flexible enough to allow for additional covariates such as batch effects or biological information that can be inferred from the gene expression (cell cycle). In addition to simulation studies that demonstrated robustness, Clonealign was also applied to real cancer datasets to discover novel clone-specific dysregulated biological pathways.

MUSIC [46] is an unsupervised topic modeling method for integrative analysis of single-cell RNA data and pooled clustered regularly interspaced short palindromic repeats (CRISPR) screening data [47]. The model links the gene expression profile of the cells and specific biological functions by delineating perturbation effects, allowing for a better understanding of perturbation functions in single-cell CRISPR data. In the perturbation effect prioritizing step, MUSIC utilizes the output from the topic model and estimates individual gene perturbation effects on cell phenotypes. It takes three different schemes in modeling the gene perturbation effect in combined single-cell and CRISPR data: an overall perturbation effect, functional topic-specific perturbation effects represented by a topic model, and relationships between different perturbation effects. MUSIC was applied to 14 real single-cell CRISPR screening datasets and accurately quantified and prioritized the individual gene perturbation effect on cell phenotypes, with tolerance for substantial noise.

### Manifold alignment methods

Manifold alignment methods aim to infer a lower-dimensional structure within multiple complex datasets (Figure 3B). Once this is done, points can be matched across the datasets. This is a very broad class of algorithms, and we here review several representative ones based on distinct ideas, such as the use of pseudotime trajectories, Kernel methods, and distance-based matching of cells.

MATCHER [48] is the first manifold alignment technique to align different forms of single-cell data. Their approach builds on trajectory inference [49]. It constructs pseudotime trajectories corresponding to cellular processes for each omic first and then aligns them between different omics. Pseudotime trajectory models the corresponding cellular process as a Gaussian process and infers the latent variable corresponding to pseudotime. This results in a set of curves capturing the biological processes, one for each omics layer. Such curves are then projected onto a reference line so that different cells can be matched across omics. The model makes a strong assumption that there is only one common biological process to be modeled.

Maximum mean discrepancy-based manifold alignment (MMD-MA) [50] is a completely unsupervised method. The alignment is performed by matching low-dimensional representations of different omics, constructed through a kernel-based technique that minimizes the maximum mean discrepancy (MMD) [51] between the two datasets. Additionally, the representations are constructed by taking into account the distortion of the distances in the original data while keeping the transformation as simple as possible. The model was evaluated on data containing gene expression and methylation values from the same single cells; the known cell correspondence information was hidden, and MMD-MA was able to successfully reconstruct this information.

**Figure 4:**
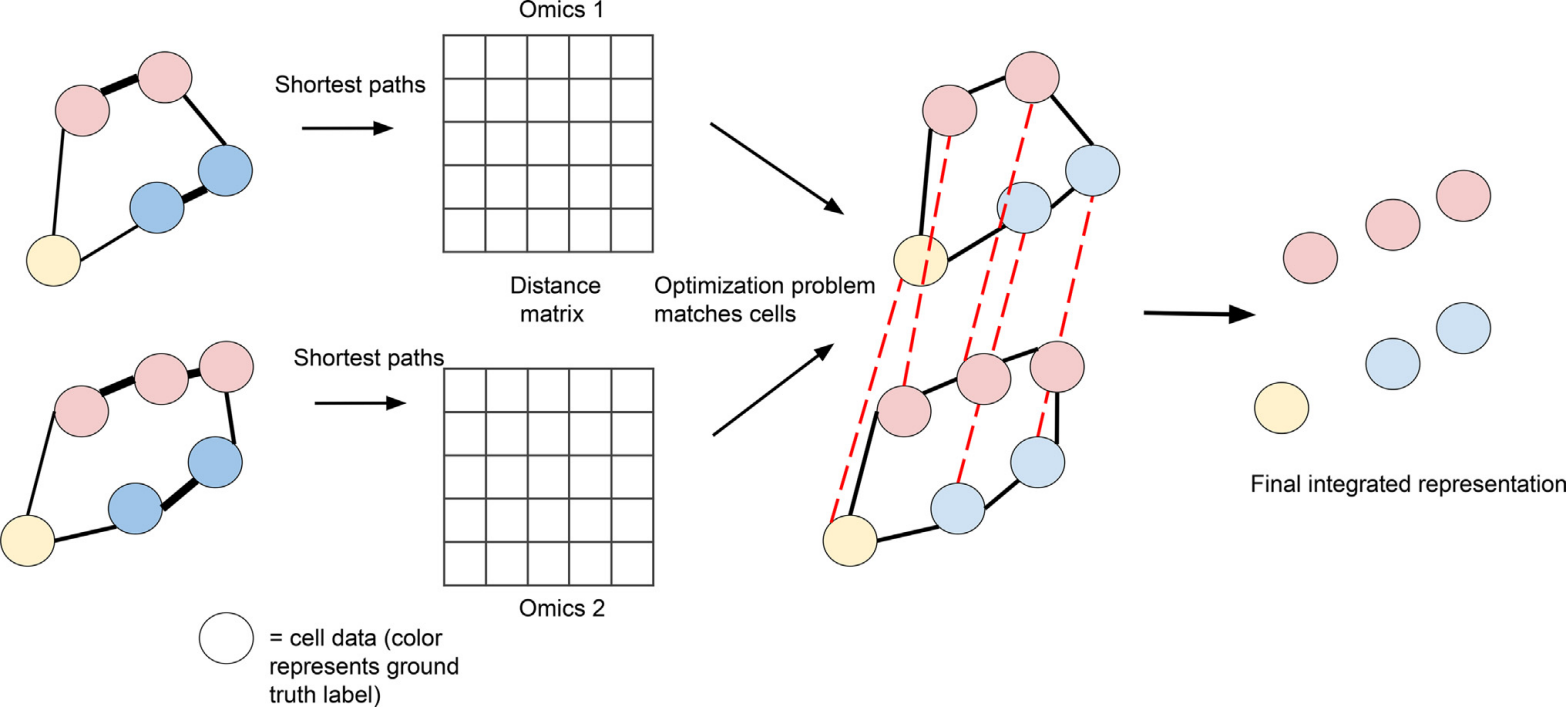
**Distance-based alignment** Schematic overview of the distance-based alignment algorithm: cells are represented by nodes in two different graph representations, corresponding to two different omics assays. Cells with very similar omics measurements are connected to form graphs. Two graphs are then aligned in order to preserve a notion of distance on the graph.

Distance-based matching methods represent a class of methods performing unsupervised alignment of different omics datasets by matching the structure of the datasets (Figure 4). Representative methods include UNION-Com [52], SCOT [53], and Pamona [53]. Their common idea is that if different omics layers indeed correspond to similar samples of cells, then the distance matrices of any two omics layers will become very similar after rearranging the cell indices. Distances between cells from different omics are computed by considering kNN graphs in different omics layers and finding the nearest distance along the graph. UNION-Com [52] finds a matching matrix connecting points across datasets by optimizing the similarity of distance matrices after cell permutation. This approach of matching is an extension of generalized unsupervised manifold alignment (GUMA) [54] with newly allowed soft matching. Subsequently, this method performs a version of *t*-distributed stochastic neighbor embedding (*t*-SNE) [55] adopted for multi-modal data represented in the same latent space. SCOT [53] performs soft matching via a different optimization problem per the theory of optimal transport. The quantity minimized is the Gromov-Wasserstein distance, which generalizes the earth-mover Wasserstein distance to optimal transport between different spaces [56]. Pamona [57] uses a similar approach to SCOT, but with a modification of optimal transport based on partial Gromov-Wasserstein distance [58], which accounts for data points that do not have appropriate matches across datasets. By doing so, it allows for possible imperfect alignment between the datasets, tolerating cell types present in one dataset only. After the alignment is found, the data corresponding to different modalities are projected down to a dimensionally reduced space using Laplacian eigenmaps [59]. This approach manages to take the overall structure of all datasets into account while matching the cells, without the requirement of identical distributions of different modalities.

Limited benchmarking was performed in the original studies in the distance-based matching methods. UNION-Com compared favorably with Seurat v3 and MMD-MA when evaluated on the quality of labels transferred between gene expression, methylation, and chromatin accessibility data [52]. SCOT compared favorably to MMD-MA and UNION-Com on several real and simulated datasets containing transcriptomic and epigenetic (DNAme or chromatin accessibility) data [53]. Pamona outperformed SCOT, MMD-MA, and Seurat v3 [57], when benchmarked on several datasets containing transcriptomic and epigenetic data. Clearly, more comprehensive comparison is needed to evaluate this class of methods over other modeling approaches.

### Neural network-based methods

Neural networks, including autoencoders and generative adversarial networks (GAN), have been used for the unsupervised task of the alignment of omics datasets. Autoencoders have been described earlier. GANs typically consist of two parts: the generator network and the discriminator network. The generator tries to produce outputs of a form resembling a certain target dataset, and the discriminator is optimized to learn the difference between the generator’s outputs and the elements of the target dataset. In this section, we summarize the relevant neural network methods below.

SCIM [60] builds on a multi-domain translation approach [61] to integrate multi-omics data in an unsupervised fashion. It uses a separate variational autoencoder for each modality in order to map the data onto reduced latent space representations. Such representations are then aligned to have a similar structure, by using a discriminator network in addition to autoencoders which learns to distinguish between the latent space representations of different omics. The two autoencoders and the discriminator network are trained simultaneously, resulting in the two latent spaces being maximally alike. Once both datasets are encoded into approximately corresponding representations, the points with similar latent representations are matched across the datasets. This model was tested on simulations from probabilistic simulation of single-cell RNA-seq tree-like topologies (PROSSTT) [62] as well as datasets containing gene expression and proteins and performed favorably to MATCHER when applied to simulated data exhibiting a complex cellular differentiation process.

MULTIGRATE [63] uses a multi-modal variational autoencoder structure to project multi-omics data onto a shared latent space. Although somewhat similar to the scMVAE model [28], this framework brings additional flexibility and can be used for the integration of paired and unpaired single-cell data. Furthermore, this model can integrate data from a multi-omics assay such as CITE-seq with data from a single-omics assay such as scRNA-seq. Data corresponding to different omics are first passed through separate neural networks, before being combined by the product of experts technique [29] to form the latent distribution. The decoder networks then aim to reconstruct all of the omics from this unified representation. To better align cells, MMD is added to the loss function, penalizing the misalignment between the point clouds belonging to different assays. Their model was used for the creation of multi-modal atlases, and mapping of a COVID-19 (coronavirus disease of 2019) single-cell dataset onto a multi-modal reference.

MAGAN [64] utilizes GANs to align data from different domains. MAGAN uses two tied GANs to translate between the omics layers, while tying their parameters and requiring that their combination maps any point onto itself. Namely, if the first generator maps data point A to data point B, then the second generator should map B back to A. It is conceptually very similar to the CycleGAN [65] model from computer vision, but with a key innovation that allowed it to more efficiently align and integrate single-cell data. The novelty here was noting that while the CycleGAN framework was very good at aligning the datasets in aggregate, it would not necessarily correctly match individual points. This is a particularly important problem for single-cell data. To address this problem, MAGAN is augmented with a correspondence loss measuring the difference between points before and after being mapped by generators. This model was tested on a variety of datasets, ranging from a simulated dataset to Modified National Institute of Standards and Technology (MNIST) handwritten digits to molecular data. The method was applied to combine transcriptomic and proteomic data in single cells. The model was shown to meaningfully align the datasets even when the correspondence information was not available.

### Other methods

Some of the methods previously developed for aligning different scRNA-seq datasets, could in principle be repurposed for single-cell multiple omics alignment as well. In such cases, different omics data are aggregated over genes and converted into gene activity scores, sharing the same format with scRNA-seq data. Here we cover two of such methods, LIGER and Seurat, due to their wide popularity. A caveat of this approach is the lack of the ability to individually model the omics data. Due to the space limitation, we recommend readers to earlier benchmark studies [66] on other scRNA-seq integration methods.

Canonical correlation analysis (CCA) based methods reduce the dimensionality of data by selecting the degrees of freedom that are correlated between the datasets**.** Seurat v3 [67] combines CCA with network concepts in order to align and integrate single-cell multi-omics data. After performing the CCA, the algorithm identifies anchors between the datasets and scores the quality of those anchors. Anchors are identified by mutual nearest neighbors (MMNs), and their quality is scored by considering the overlap between the neighborhoods of anchors. Similar to Seurat v3, MAESTRO [68] also utilized canonical correlation analysis for the integration of transcriptomic and epigenetic data and provided a comprehensive analysis pipeline. bindSC [69] also uses canonical correlation analysis to construct shared representations of the data, iteratively optimized using a custom procedure.

LIGER [70] performs an integrative non-negative matrix factorization (iNMF) to learn factors explaining the variation within and across datasets. Data such as DNA methylation are first aggregated over genes. Cells corresponding to different datasets are described by separate sets of cell-specific factors. Gene factors consist of two components: one that is shared across datasets and one that is dataset specific; the model aims to make the dataset-specific portion as small as possible. After performing the matrix factorization, the shared factor neighborhood graph is formed, in which cells are connected based on the similarity of their factors and used for aligning the cells across modalities. Recently, this nonnegative matrix factorization approach has been extended to incorporate the idea of online learning. It iteratively updates the model in real-time and leads to better scalability and computational efficiency [71].

## Concluding remarks

The landscape of experimental techniques for omics sequencing and analyzing the data has grown significantly in the last few years. Accompanying the thrust of technological advancement, an increasing body of computational methods to handle multi-omics data integration or alignment have been proposed. Geared toward computational biologists and genomics scientists, here we reviewed in-depth and extensively these computational methods by their working principles. Among these methods, AI and machine learning-based methods account for the majority, demonstrating the influence in single-cell computational biology. Other approaches using matrix factorization and Bayiean’s methods have also been proposed. As demonstrated in a range of methods, the integration of multi-omics data at the single-cell level improves the quality of downstream biological interpretation steps, such as clustering. With the advent of technologies for sequencing multi-omics data from the same single cells, efficient multi-omics integration methods to provide further biological and medical insights at larger scales will be in continued demand.

Meanwhile, the rapidly growing number of computational methods pose an urgent need for benchmarking studies on their performances, in order to provide guidelines to choose appropriate methods for specific datasets. Current comparisons are either incomplete or using a small set of benchmark datasets, with inconsistent metrics in various studies, impeding the selection of appropriate methods for the dataset to analyze. This is made more difficult by the generally unsupervised nature of the integration task, where commonly required ground truths are not known for certain. Moreover, different methods have different prerequisites regarding preprocessing steps, normalization, *etc*., and as a result, careful consideration of these steps and their impacts on the model performances is needed. Oftentimes, the integration methods were developed with one specific application/assay in mind, generalization of these methods with the emergence of new technologies needs to be demonstrated. Fortunately, some benchmarking studies have been conducted in other sub-fields of single-cell computational biology for references, such as those focused on the integration of data from different cells and atlas study [72], cell-type annotation [73], and integration algorithms for spatial transcriptomics [74]. Creating standardized high-quality benchmarking datasets would aid such efforts, as proposed in [75] for scRNA-seq data. Such datasets should have a well-defined ground truth, ideally confirmed by expert annotation and supported by previous literature. One promising candidate for this benchmark dataset has been proposed by open problems in single-cell analysis competition at the NeurIPS conference [76]. It is always a good idea to complement the real datasets with simulated datasets, as biases may exist in the ground truth. Although multi-omics simulation datasets specifically designed for single cells are missing, one can nevertheless modify some simulation tools previously designed for bulk tissues [77]. Finally, comprehensive and flexible benchmarking pipelines that can accommodate the ever-increasing body of integration methods will be extremely useful, in keeping the field up-to-date on multi-omics integration. One such example is the dynverse [78].

Due to the scope, one area that we did not cover in this review is the integration between single-cell omics with other modalities of data. For example, integrative analysis between scRNA-seq and spatial omics data [79], [80], [81], [82], [83] and imaging data (*e.g.*, hematoxilin and eosin stain, or H&E image). In the data types with spatial measurements, the spatial relationships need to be considered in addition to the feature matrix. We refer readers to other recent reviews covering this area [84], [85]. Given that the multi-omics integration and alignment computational research is a thriving area, we have created an open review document online using the manubot protocol (https://github.com/lanagarmire/multiomics_review_manubot; accessed on July 18, 2022). We encourage the community to contribute to this open document to keep the review up-to-date.

## CRediT author statement

**Stefan Stanojevic:** Writing - original draft, Writing - review & editing. **Yijun Li:** Writing - original draft, Writing - review & editing. **Aleksandar Ristivojevic:** Writing - review & editing. **Lana X. Garmire:** Supervision, Writing - review & editing. All authors have read and approved the final manuscript.

## Competing interests

The authors have declared no competing interests.
